# Accelerated Tooth Movement with Orthodontic Mini-Screws

**DOI:** 10.1155/2017/2327591

**Published:** 2017-12-14

**Authors:** S. Aksakalli, A. Balaban, K. Nazaroglu, E. Saglam

**Affiliations:** ^1^Department of Orthodontics, Istanbul Aydin University, Istanbul, Turkey; ^2^Department of Orthodontics, Bezmialem Vakif University, Istanbul, Turkey; ^3^Periodent Dental Clinic, Istanbul, Turkey; ^4^Department of Periodontology, Bezmialem Vakif University, Istanbul, Turkey

## Abstract

This case report outlines the possibility of accelerated tooth movement with the combination of microosteoperforation and mini-screws. A 14-year-old male patient presented Class II malocclusion with maxillary incisor protrusion. Upper first premolars were extracted, and after leveling, accelerated canine distalization started. For pre- and postdistalization times, amount of distalization, periodontal health, and root resorption were assessed. Within the limitations of this case report, micro-osteoperforations with mini-screw have a potential for shortening the treatment time.

## 1. Introduction

Reducing the duration of orthodontic treatment is still challenging. It is one of the objectives of both orthodontists and patients because long treatment may cause caries, root resorption, gingival recession, and time loss [1].

To achieve faster results, several attempts have been applied such as piezocision [2], micro-osteoperforations (MOPs) [3], laser [4], and vibration [5], and these attempts can be classified under the following categories: drugs, surgical methods, and physical/mechanical stimulation methods. But, there are lots of unanswered questions towards most of these attempts.

Micro-osteoperforation technique has been found successful both on humans and animals [6]. In this technique, the inflammatory marker levels are increased, and this situation leads to increased osteoclastic activity and velocity of tooth movement. The rate of canine retraction increased 2.3-fold compared with the control group, when MOP was performed with a device called Propel (Ossining, NY) [3]. In this case report, we compared the amount of canine retraction of MOP with mini-screws and the effect of MOP on periodontal health.

## 2. Case Presentation

A male aged 14 years presented complaining of forward placement of his upper teeth and upper lip. The patient had convex profile and incompetent lips.

The maxillary incisors were proclined, and the overjet was 3.3 mm. The molar and canine relationships were Class II on both sides. Maxillary midline was on the right side 2.5 mm, and mandibular midline was on with the facial midline. The oral hygiene was satisfactory.

The panoramic radiograph confirmed the presence of all permanent teeth. Bone levels were normal. Cephalometric analysis revealed skeletal Class II pattern with maxillary prognathism. There were no periodontal or systemic illness, poor oral hygiene, and alveolar bone loss.

Our objectives were to establish Class II molar and Class I canine relationship, improve facial esthetics, gain lip competence, and obtain ideal overjet and overbite.

After ethical approval and case discussion, upper premolars were extracted, and Nance appliance was placed to reinforce the anchorage. The patient was educated and informed about oral hygiene in orthodontics. Roth's prescription edgewise brackets (Master Series, American Orthodontics, Sheboygan, WI) with 0.018-inch slots were used. After leveling and alignment stage, 16 × 22 stainless steel wires were placed for maxillary teeth. To distalize canines, 120 gr closed coils (GAC International) were used for both sides. The study involved a split-mouth design. MOP was applied for one side with mini-screws, and the other side was control side.

**Figure 1:**
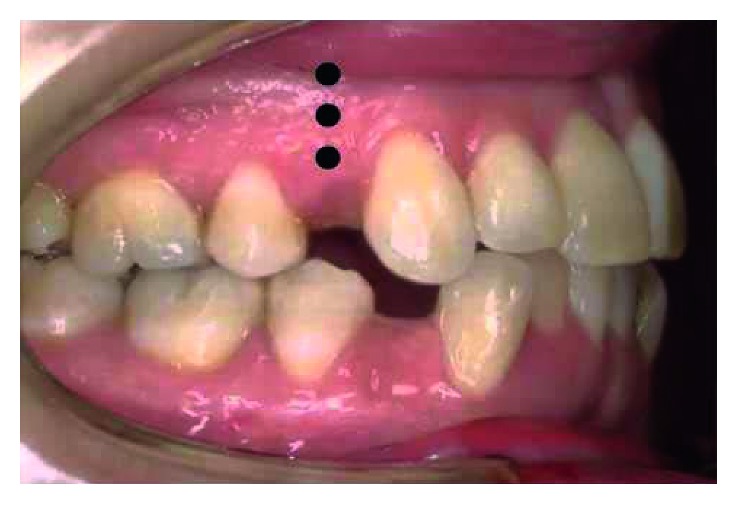
Experimental model. Black dots show MOP areas.

**Figure 2:**
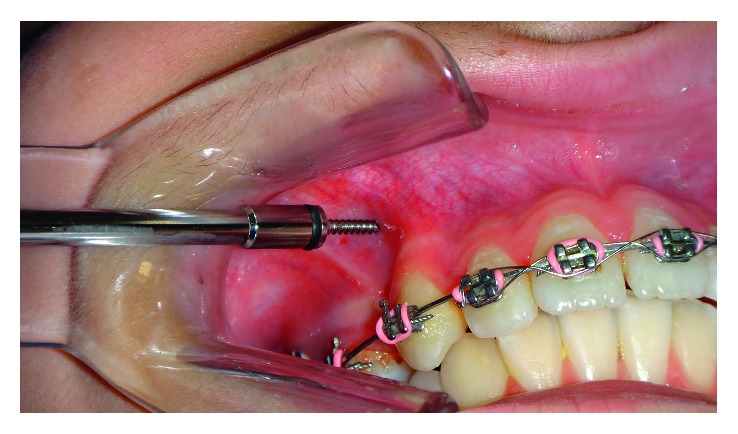
Application of MOP with mini-screw.

MOP was applied as described by Alikhani et al. [3]. Three MOPs were applied distal to the canines (Figure 1) and before canine distalization using mini-screws of 8 mm length and 1.5 mm diameter (Orlus, Henry Schein Orthodontics); each perforation was 1.5 mm wide and 5 mm deep (Figure 2). Mini-screws were inserted with handpiece and carried to the mouth without being touched by the hand. MOPs were performed under local anesthesia (2% lidocaine with 1 : 100,000 epinephrine). Any flap operation was not applied, and antibiotic or pain medication was not prescribed.

**Figure 3:**
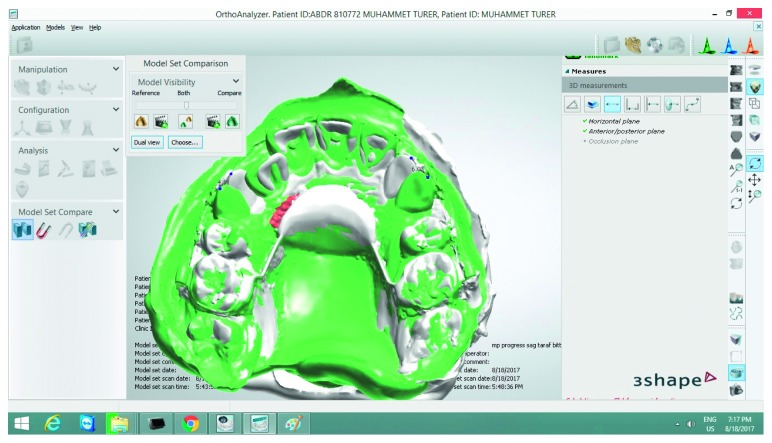
3D superimposition of models.

The patient was examined in 2-week intervals, and the distalization forces were checked until Class I canine was established. Before (T0) and after (T1) distalization, maxillary models were scanned in a 3shape R900 model scanner (3shape, Copenhagen, Denmark) (Figure 3). Models were superimposed, and distalizations of the canines were measured as described in a similar study [2]. To check the health status and the success of the MOPS, gingival index, probing depth, and mobility status were measured. For mobility, Muhlemann index was used [7]. Silness and Loe [8] index was selected for periodontal status. Radiographic assessments were done at T0 and T1.

**Figure 4:**
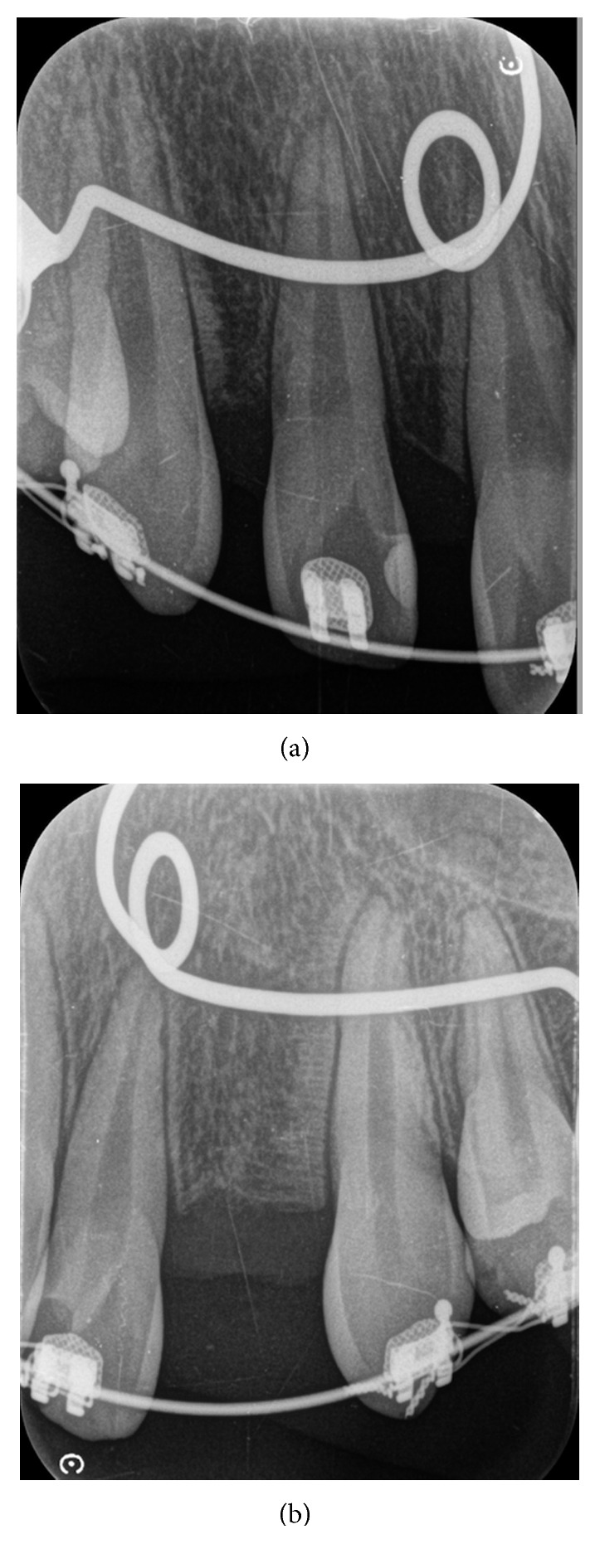
Periapical radiographs at T1. (a) Control side; (b) MOP side.

**Figure 5:**
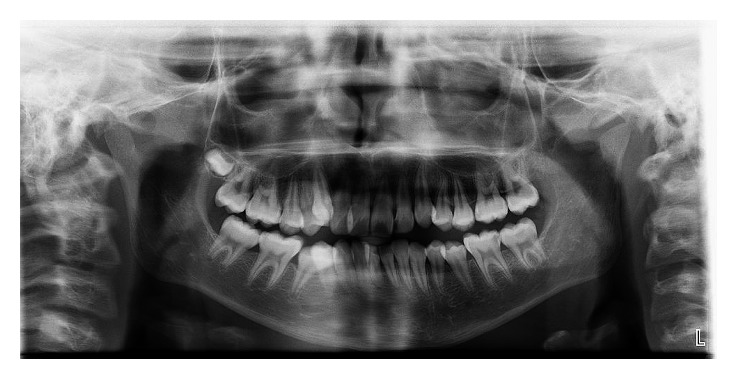
Panoramic radiograph at T0.

According to 3D analysis, canine distalization was 6.03 mm in MOP side after 91 days, whereas it was 4.11 mm in control side (Table 1). Gingival index and bleeding in probing were decreased; probing depth was not changed after distalization, whereas mobility scores were increased (Table 2). There is no root resorption for both upper canines (Figure 4). Additionally, there was no root resorption at T0 (Figure 5).

## 3. Discussion

Several methods have been studied, and acceptable results were gained by these rapid tooth movement methods. Rapid orthodontic tooth movement was studied with injection of prostoglandins [9], osteocalcin [10], etc. These methods are biochemical in nature and hard to prepare and apply. Piezocision was successful in canine retraction [2]. In this method, clinician needs piezosurgery unit, and vertical interproximal incisions have to be performed before piezoincision cuts. Low-level laser therapy was found successful for accelerated tooth movement by Fujita et al. [11]. For this method, a laser unit is needed, and contradictory results were found in the literature against this method [1]. Another rapid tooth movement method, interseptal bone reduction or corticotomy [12], was more traumatic and invasive methods than MOP. Cheung et al. [13] stated that mini-screw-facilitated MOPs could effectively accelerate tooth movement in rats. Additionally, Alikhani et al. accelerated tooth movement in humans with MOP method by using a Propel device (Ossining, NY). In this case report, we tried to combine MOP method with mini-screws to accelerate canine distalization in the light of these informations. It was stated that MOP with a Propel device could increase the rate of canine retraction by more than twofold. In this case report, MOP with mini-screws increase the rate of retraction by almost 1.5-fold. It was thought that MOP method increased tooth movement by inducing more rapid bone remodeling and also increased osteoclast quantity and new bone formation on MOP sides [3]. It has been shown that increasing the number of perforations can increase the level of cytokines and osteoclastogenesis that can increase the rate of tooth movement [6]. So, clinicians should select the number of the MOPs case by cases and not limit themselves to 3.

Mini-screws, sometimes referred to as “temporary anchorage devices,” are widely accepted in the treatment of malocclusions. Over the last century, anchorage support was obtained from other teeth, the palate, alveolar ridges, or appliances. By the development of mini-screws, new dimensions for anchorage were found. The volume of the publications about mini-screws is expanding, but still scientific researches are needed such as controlled trials or histological examinations [14]. Orthodontists continue to study or report cases about different applications of mini-screws [15]. Although generally they are used for temporary anchorage units, in this case we use them as a unit for accelerating tooth movement.

Many factors could affect the rate of tooth movement. The type of tooth movement is one of these factors [16], so we tried to distalize canines bodily with stainless steel wire and closed-coil combination. Occlusal forces can also affect the rate of movement [17]. To prevent this effect, patients with crossbite or deviation during closure were excluded. Patients with poor oral hygiene, bone loss, and periodontal or systemic disease were excluded.

Periodontal status and root resorption are question marks for accelerated teeth. In this case report, probing depth, gingival index, and bleeding on probing scores did not change at T1 except mobility score. Root resorption was assessed by panoramic radiographs and a periapical radiograph at T1. There is no root resorption at T1.

Accelerated tooth movement methods had uncertainties and some disadvantages that made them not commonly used now. But a rapid increase in the interest of clinicians, companies, and patients will lead us the best results. Within the limitations of this case report, MOP method with mini-screws accelerated canine distalization by showing no harmful effects on root and periodontal structures.

## Figures and Tables

**Table 1:** Amount of canine distalization every month.

	1st month	2nd month	3rd month	Total
MOP side	2.35 mm	2.02 mm	1.66 mm	6.03 mm
Control side	1.20 mm	2.01 mm	0.90 mm	4.11 mm

**Table 2:** Periodontal health of the canines.

		Mobility score	Gingival index	Probing depth
MOP side	T0	1	1	2 mm
	T1	2	1	2 mm
Control side	T0	1	1	2 mm
	T1	2	1	2 mm
